# Patterns of lifestyle risk behaviors for cardiovascular disease in family caregivers: a latent class analysis

**DOI:** 10.3389/fpubh.2025.1593898

**Published:** 2025-06-17

**Authors:** Soojung Ahn, Elisa H. Son, Mulubrhan F. Mogos, James M. Muchira, Ying Sheng, Chorong Park, Lena J. Lee

**Affiliations:** ^1^Connell School of Nursing, Boston College, Chestnut Hill, MA, United States; ^2^Translational Biobehavioral and Health Promotion, National Institutes of Health Clinical Center, Bethesda, MD, United States; ^3^School of Nursing, Vanderbilt University, Nashville, TN, United States; ^4^College of Nursing, Seoul National University, Seoul, Republic of Korea

**Keywords:** health risk behaviors, health promotion, cardiovascular diseases, caregivers, latent class analysis

## Abstract

**Introduction:**

Lifestyle risk behaviors for cardiovascular disease (CVD) often co-occur. However, little is known about their co-occurrence patterns among family caregivers, a high-risk population for CVD. This study aimed to identify distinct latent classes of lifestyle risk behaviors for CVD among caregivers and to examine socio-demographic, health-related, and caregiving characteristics associated with membership in the latent classes.

**Methods:**

We conducted a cross-sectional secondary data analysis of the 2019 Health Information National Trends Survey 5 Cycle 3, involving 643 unpaid family caregivers in the United States. The lifestyle risk behaviors for CVD included current cigarette use, current alcohol consumption, low physical activity, prolonged sedentary time, low fruit intake, and low vegetable intake, as defined by established guidelines. We performed latent class analysis to identify unobserved subgroups based on these multiple lifestyle risk behaviors. Subsequently, we conducted multinomial logistic regression to investigate socio-demographic, health-related, and caregiving characteristics associated with latent class membership.

**Results:**

The majority of participants were females (55.3%) and non-Hispanic white (57.1%), with a mean age of 55 ± 16 years. Three distinct classes were identified: Class 1 (*Physically active caregivers,* 17.1%), Class 2 (*Physically inactive, healthy eaters,* 18.8%), and Class 3 (*Physically inactive, unhealthy eaters,* 64.1%). In unadjusted models, older caregivers (≥65 years) were more likely to belong to Class 2, relative to Class 1, compared to those aged 18–49 years. Caregivers with perceived financial difficulties, psychological distress, low self-efficacy in health management, and poor sleep quality were more likely to belong to Class 3, rather than Class 1, compared to their counterparts. Additionally, dementia care and caregiving ≥ 20 h/week were significantly associated with Class 3 membership. In the adjusted model, psychological distress remained significant. Caregivers reporting psychological distress were more likely to belong to Class 3 rather than Class 1, compared to those without psychological distress.

**Conclusion:**

Our findings reveal the presence of subgroups of caregivers with unique patterns of lifestyle risk behaviors, with most not meeting the recommended levels of health behaviors. Future studies should consider these co-occurring patterns along with the key factors associated with higher-risk lifestyle behavior patterns when developing interventions to promote caregivers’ cardiovascular health.

## 1 Introduction

The health impact of caregiving represents a significant public health concern, affecting a substantial portion of the population. Currently, nearly 20% of Americans provide unpaid care to family members or significant others with health or functional needs (1). Although caregiving can be fulfilling and rewarding, it can also be extremely demanding, particularly when it involves prolonged and intense care, leading to considerable emotional, physical, and financial strain. Accumulating evidence indicates a link between caregiving and increased risks of cardiovascular disease (CVD) (2, 3). While the mechanisms underlying this connection are not fully elucidated, it is hypothesized that caregivers’ limited engagement in healthy lifestyle behaviors, constrained by time, emotional and physical burden, may contribute to this increased risk (4, 5). Caregivers are more likely to encounter lifestyle challenges, such as insufficient exercise or physical activity, sleep disturbances, changes in diet and eating habits, alcohol consumption, and cigarette use, compared to non-caregivers (6–9).

Lifestyle risk behaviors frequently co-occur, with evidence suggesting that a significant proportion (≥60%) of the population engages in multiple risk behaviors (10–13). For instance, physically inactive individuals are also likely to be current smokers and report heavy alcohol consumption and poor dietary quality (10, 14, 15). These behaviors are often interrelated and may exert a synergistic effect on chronic illness and mortality (16, 17). Consequently, targeting a single behavior in an intervention may be less effective than addressing co-occurring behaviors to achieve favorable health outcomes, such as improved cardiovascular health (18, 19). Moreover, the co-occurrence patterns of lifestyle risk behaviors are unlikely to be uniform within a population. Various factors, including demographic, health- or clinical-related, and social factors, can influence the patterns. For example, older women tend to have healthier lifestyle profiles compared to young male adults, and socio-economically disadvantaged groups are more likely to exhibit multiple lifestyle risk behavior patterns than their counterparts with advantageous socio-economic status (20, 21). Psychological health and quality of life were also associated with different health behavior patterns in the general population (14, 22). Thus, subgroups of a population may exhibit distinct configurations of lifestyle risk behaviors.

Understanding the co-occurrence of multiple lifestyle behaviors in a population and the factors associated with these patterns is crucial for developing targeted and effective interventions. Instead of focusing on a single lifestyle risk behavior, such interventions can address a group of lifestyle risk behaviors. While there have been studies on patterns of health behaviors in various populations, including adolescents, middle-aged women, and cancer survivors (23–25), little is known about the co-occurrence of lifestyle risk behaviors among caregivers (26). Given the unique circumstances in which caregivers are prone to various lifestyle challenges and the burdens of caregiving, it is critical to better understand the health behavior patterns and the characteristics of those at higher risk of multiple lifestyle risk behaviors among caregivers. Latent class analysis (LCA) is particularly suited for this purpose as it allows for the identification of distinct subgroups within a population based on their co-occurring behaviors. LCA accounts for the interrelationships among variables to identify subgroups of individuals with similar characteristics, potentially providing an advantage over univariate methods (27). This method may also show a realistic picture of how caregivers engage in lifestyle risk behaviors in their daily lives. To address the knowledge gap in the caregiving literature, therefore, the aims of this study were (1) to identify co-occurrence patterns of lifestyle risk behaviors (i.e., current cigarette use, current alcohol consumption, low physical activity, prolonged sedentary time, low fruit intake, and low vegetable intake) among caregivers using LCA and (2) to investigate sociodemographic, health-related, and caregiving-related factors associated with membership in the identified latent classes.

## 2 Methods

### 2.1 Study sample

This study is a cross-sectional secondary analysis utilizing data from the 2019 Health Information National Trends Survey (HINTS), Cycle 3, conducted between January 22 and May 7, 2019. This survey, administered by the National Cancer Institute, is designed to gather information on how non-institutionalized adults in the U.S. use cancer-related information (28). Detailed survey design, sampling and weighting processes are documented in the HINTS 5 Cycle 3 methodology report (29). Briefly, the sample selection procedure involved a two-stage design: first, a stratified sample of addresses was selected from a database of residential addresses using an equal-probability sample. Second, one adult was chosen from each sampled household by the respondents themselves (29). The sampling frame included all non-vacant residential addresses in the U.S., with oversampling in high-minority areas where the population proportion of Hispanics or African Americans was ≥ 34% to increase precision for minority subpopulations (29). The survey was conducted through mailed paper surveys or web-based surveys, using an identical protocol. The overall weighted response rate for the survey was 30.3%. Each iteration of HINTS underwent expedited review approved by the Westat Institutional Review Board and was classified as “not human subjects research” by the National Institutes of Health Office of Human Subjects Research.

**Figure 1:**
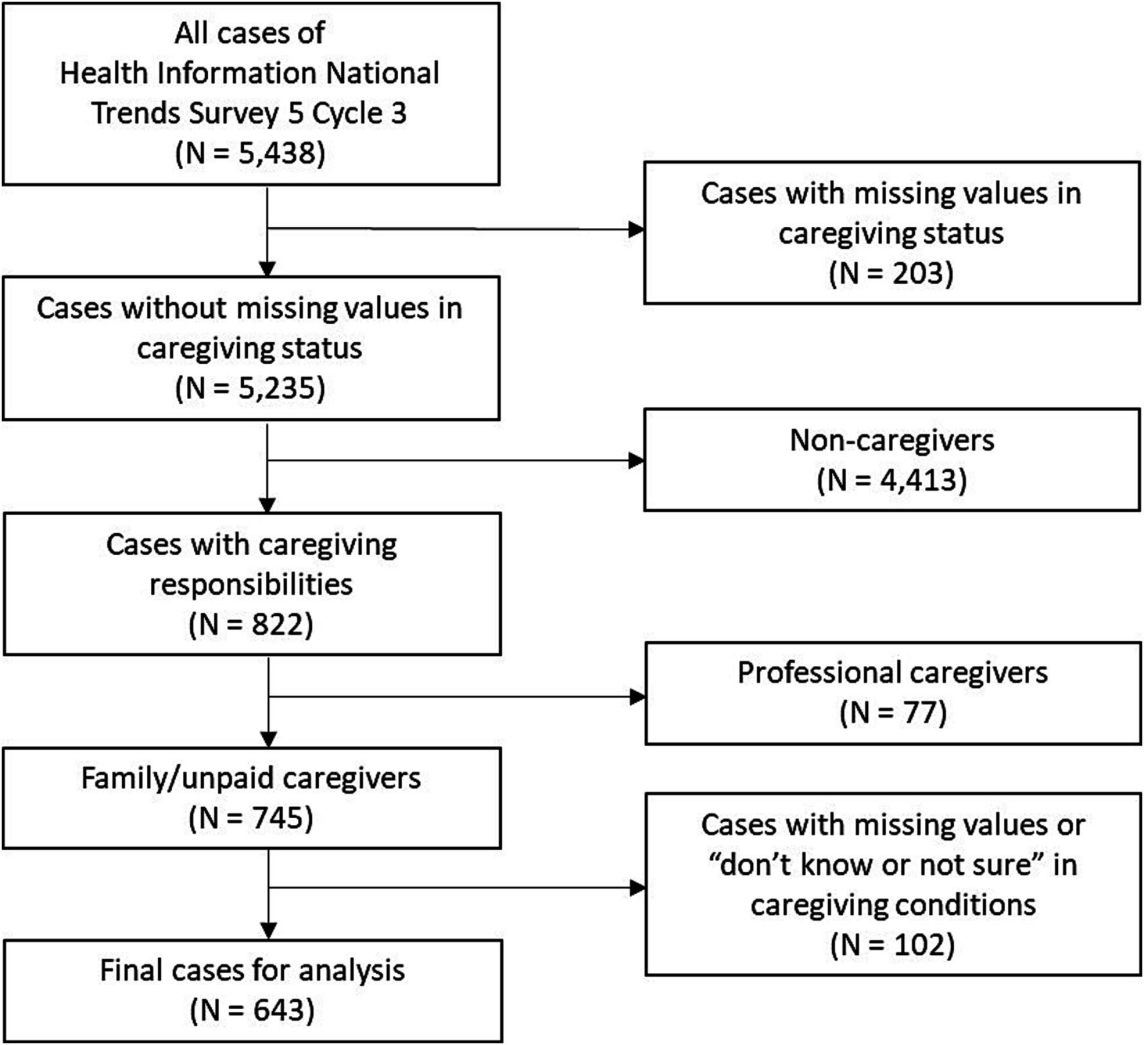
Sample selection flow.

Our sample selection is illustrated in Figure 1. In this study, we focused on respondents who identified themselves as caregivers. Of the total HINTS 5 Cycle 3 sample (*N* = 5,438), there were 203 cases with missing values in caregiving status, and 4,413 respondents indicated they were not caregivers. A total of 822 (15.1%) reported that “[they] are currently caring for or making healthcare decisions for someone with a medical, behavioral, disability, or other condition,” specifying the relationship of the care-recipient to them (i.e., child, spouse/partner, parent(s), another family member, or friend/non-relative). To more accurately identify family/informal/unpaid caregivers who were significantly engaged in care, we excluded 77 respondents who provided care as part of a job and 102 respondents who did not know the care-recipient’s main health conditions or did not respond. Ultimately, we included 643 caregivers in our analytic sample for this study (weighted *N* = 30,253,974).

### 2.2 Measures

#### 2.2.1 Lifestyle risk behaviors

##### 2.2.1.1 Current cigarette use

Respondents were asked if they had smoked at least 100 cigarettes in their entire life and about their smoking frequency. Based on the responses, cigarette use status was categorized as current vs. never/former user. The use of e-cigarettes was also included.

##### 2.2.1.2 Current alcohol consumption

Respondents reported the number of days per week they consumed at least one alcoholic drink in the past month and the average number of drinks on those days. Using the number of average drinks per week, alcohol consumption was categorized as current drinker (≥1 drink per week) vs. non-drinker (<1 drink per week). Despite the mixed nature of evidence on the health benefits of moderate drinking, we decided to treat current alcohol consumption as a risky behavior following the 2020–2025 Dietary Guidelines for Americans (30). The guidelines recommend that adults who do not drink should not start drinking for any reason, and those who do drink should do so in moderation by limiting intake. However, the guidelines also presented emerging evidence suggesting that even drinking within the recommended limits may still increase the overall risk of death (30). Additionally, a recent meta-analysis study did not show a significantly beneficial association between low to moderate alcohol consumption and reduced risk of all-cause mortality (31).

##### 2.2.1.3 Low physical activity

Respondents indicated the number of days per week they engaged in moderate-intensity physical activity or exercise and the duration of these activities. They also reported the frequency of leisure-time activities for strength training outside of their job. Based on the Physical Activity Guidelines for Americans (moderate exercise≥150 min/week AND strength training≥2 days/week), respondents were categorized into two groups: those not meeting the recommended levels of physical activity (low physical activity) vs. those meeting the recommendation (32).

##### 2.2.1.4 Prolonged sedentary time

Respondents reported their daily sitting time at home or at work. Sedentary behavior was categorized as ≥8 h (prolonged sedentary time) vs. <8 h per day, based on meta-analysis studies that reported the dose–response relationship between sedentary behavior and all-cause and CVD mortality, adjusted for physical activity (33, 34).

##### 2.2.1.5 Low fruit intake

Respondents indicated their daily fruit intake, including 100% pure fruit juice, using the response options ranging from none to 4 or more cups. Based on the 2020–2025 Dietary Guidelines for Americans (≥1–2 cups/day), respondents were categorized into a group not meeting the fruit consumption recommendation (low fruit intake) vs. a group meeting the recommendation (30).

##### 2.2.1.6 Low vegetable intake

Respondents reported their daily vegetable intake, including 100% pure vegetable juice, using the same response options as fruit consumption. Based on the 2020–2025 Dietary Guidelines for Americans (≥2–3 cups/day), respondents were categorized into two groups: those not meeting the recommendation (low vegetable intake) vs. those meeting the recommendation (30).

#### 2.2.2 Sociodemographic factors

Sociodemographic factors included age (18–49, 50–64, ≥65 years), sex (male, female), race and ethnicity (Hispanic, non-Hispanic Asian, non-Hispanic Black or African American, non-Hispanic other [American Indian or Alaska native, Native Hawaiian or other Pacific Islander, multiple races mentioned], non-Hispanic White as pre-specified in the survey data), education (<college [less than high school, high school graduate, some college], ≥college educated [college graduate, postgraduate]), marital status (married or partnered, not married or partnered [single, divorced, widowed, separated]), household income (<$50,000, $50,000–$99,999, ≥$100,000), perceived financial status (living comfortably, getting by, finding it difficult/very difficult on present income), and rural–urban status by the Rural–Urban Continuum Code per the US Department of Agriculture Economic Research Service (35) as informed by a previous study (36) (rural, urban areas).

#### 2.2.3 Health-related factors

Health-related factors included body mass index (<30 kg/m^2^, ≥30 kg/m^2^), medical conditions (composite of diabetes, hypertension, heart condition, chronic lung disease, and depression or anxiety disorder; 0–1 condition, 2–5 conditions), self-rated health status (excellent/very good/good, fair/poor), and psychological distress (Patient Health Questionnaire-4; no distress [0–2], mild to severe distress [3–12]). Self-efficacy in health management was asked using a single item, *“Overall, how confident are you about your ability to take good care of your health?”* and was dichotomized as described in a previous study (low [somewhat/a little/not confident], high [very confident/completely]) (37). Sleep duration was categorized as <7 h vs. ≥7 h per night, based on the American Academy of Sleep Medicine’s recommended sleep hours for adults (38). Sleep quality was dichotomized into poor (very bad/fairly bad) and good (fairly good/very good).

#### 2.2.4 Caregiving-related factors

Respondents reported the relationship of the care-recipient to them, the care-recipient’s health conditions (cancer; Alzheimer’s, confusion, or dementia; orthopedic/musculoskeletal issues; mental health/behavioral/substance abuse issues; chronic conditions; neurological/developmental issues; acute conditions; aging/aging-related health issues; other), and caregiving hours per week. Based on previous literature on factors associated with caregiver burden (39), the care conditions and relationships were categorized into dementia care vs. non-dementia care and spousal caregiving vs. non-spousal caregiving, respectively. Caregiving hours were categorized as <20 h vs. ≥20 h per week, as ≥20 h per week represents more intense caregiving (40).

### 2.3 Statistical analysis

For descriptive statistics of the total sample, we computed frequencies and percentages. We performed LCA using the maximum-likelihood estimation with robust standard errors to identify subgroups of caregivers sharing similar lifestyle risk behaviors. The following six indicators were included in the LCA: current cigarette use, current alcohol consumption, low physical activity, prolonged sedentary time, low fruit intake, and low vegetable intake. We referred to a set of model fit indices to determine the number of classes that best represent the patterns of lifestyle risk behaviors observed in the data. The Akaike information criterion (AIC), Bayesian information criterion (BIC), and sample-size adjusted BIC (SABIC) were used to reflect the balance between model fit and complexity, with lower values indicating better prediction. Entropy was used to assess how well the classes are separated, with values above 0.8 considered acceptable and values closer to 1 preferred (41). The Vuong–Lo–Mendell–Rubin likelihood ratio test (VLMR-LRT) and Lo–Mendell–Rubin adjusted LRT (LMR-LRT) were used to compare the fit of a k-class model to a k-1 class model. The final number of classes was determined by examining both the model fit indices and clinical interpretation.

Multinomial logistic regression analysis was conducted to identify factors associated with latent class membership. Unadjusted regression models were tested on the list of socio-demographic, health-related, and caregiving-related factors. Factors that were significant at *p* < 0.05 in the unadjusted regression models were entered into the adjusted regression model, with age and sex controlled for. Collinearity among the factors included in the model was assessed. Full information maximum likelihood was used to handle missing data in latent class indicators, whereby a survey response contributed to the LCA if data were available for at least one indicator. To address missing data in the multinomial logistic regression analysis, multiple imputation was employed. Specifically, we generated 20 imputed datasets for all socio-demographic, health-related, and caregiving-related factors with missing values (ranging from 0.6 to 9.5%). The specified estimation model was applied to each imputed dataset, and the final estimates were derived by pooling the results across all the imputed datasets. Sample characteristics by latent classes, using both the dataset with missing values and the imputed datasets, for comparisons are presented in the Supplementary Tables. Variance estimation was based on Taylor Series Linearization to account for the complex sample design of the survey. LCA was conducted using Mplus Version 8 and descriptive and multinomial logistic regression analyses were conducted using IBM SPSS Version 29.

## 3 Results

### 3.1 Sample characteristics

Most caregivers (70.4%) were aged 18–64 years, with a median age of 56 years (interquartile range, 45–66). The majority were female (59.3%), non-Hispanic White (57.1%), college-educated (53.5%), and married or partnered (66.6%). About 22% reported financial difficulties. Regarding health-related characteristics, 30.5% experienced psychological distress, 32.2% reported low self-efficacy for taking care of their health, and 14.8% rated their health status as fair or poor. Slightly more than half (50.9%) slept ≥7 h per night on average, with 73.0% rating their sleep quality good. Approximately 20% were spousal caregivers, 29% cared for individuals with dementia, and 36% spent ≥20 h per week caregiving. Other sociodemographic and health-related characteristics of the sample are presented in Table 1. The prevalence rates of lifestyle risk behaviors were as follows: 14.7% current cigarette user, 50.2% current drinkers, 75.4% low physical activity, 43.1% long sedentary time, 50.3% low fruit intake, and 71.2% low vegetable intake.

**Table 1:** Sample characteristics (*N* = 643, weighted *N* = 30,253,974).

Variable	Category	n (weighted %)
Age group, years		
	18–49	199 (31.0)
	50–64	253 (39.4)
	≥ 65	182 (28.4)
	Missing	9 (1.2)
Sex		
	Male	223 (34.7)
	Female	381 (59.3)
	Missing	39 (6.0)
Race and ethnicity		
	Hispanic	87 (13.5)
	Non-Hispanic Asian	29 (4.5)
	Non-Hispanic Black	77 (12.0)
	Non-Hispanic Other^a^	24 (3.7)
	Non-Hispanic White	367 (57.1)
	Missing	59 (9.2)
Education		
	< College	289 (45.0)
	≥ College graduate	344 (53.5)
	Missing	10 (1.5)
Marital status		
	Married/partnered	428 (66.6)
	Not married/partnered	199 (31.0)
	Missing	16 (2.4)
Household income		
	< $50,000	233 (36.2)
	$50,000 to $99,999	203 (31.6)
	≥ $100,000	198 (30.8)
	Missing	9 (1.4)
Perceived financial status		
	Living comfortably on present income	247 (38.4)
	Getting by on present income	218 (33.9)
	Finding it difficult or very difficult on present income	143 (22.3)
	Missing	35 (5.4)
Rural–urban status		
	Rural	65 (10.1)
	Urban	578 (89.9)
Body mass index		
	< 30 kg/m^2^	382 (59.4)
	≥ 30 kg/m^2^	251 (39.0)
	Missing	10 (1.6)
Medical conditions^b^		
	0–1 condition	430 (66.9)
	2–5 conditions	200 (31.1)
	Missing	15 (2.0)
Self-rated health		
	Excellent/very good/ good	544 (84.6)
	Fair/poor	95 (14.8)
	Missing	4 (0.6)
Psychological distress		
	No distress (PHQ-4 < 3)	433 (67.3)
	Mild to severe distress (PHQ-4 ≥3)	196 (30.5)
	Missing	14 (2.2)
Self-efficacy		
	Somewhat/a little/not confident (low)	190 (32.2)
	Completely/very confident (high)	453 (67.8)
Sleep duration		
	< 7 h/night	301 (46.8)
	≥ 7 h/night	327 (50.9)
	Missing	15 (2.3)
Sleep quality		
	Good	469 (73.0)
	Poor	166 (25.8)
	Missing	8 (1.2)
Dementia care		
	Yes	183 (28.5)
	No	460 (71.5)
Spousal caregiver		
	Yes	126 (19.6)
	No	517 (80.4)
Caregiving hours		
	< 20 h/week	349 (54.3)
	≥ 20 h/week	233 (36.2)
	Missing	61 (9.5)

PHQ-4, Patient Health Questionnaire-4.

a The non-Hispanic other category includes non-Hispanic American Indian or Alaska Native, Native Hawaiian or other Pacific Islander, and multiple races mentioned.

^b^ Medical conditions include diabetes, hypertension, heart condition (heart attack, angina, or congestive heart failure), chronic lung disease (asthma, emphysema, or chronic bronchitis), depression, anxiety disorder.

### 3.2 Class selection

The model fit indices for the different LCA models for lifestyle risk behaviors are presented in Table 2. A 3-class model was selected given the smallest SABIC and the entropy value greater than 0.8. In addition, the 4-class model had category probabilities below 5 to 10%, suggesting that some categories may lack sufficient representation for meaningful interpretation unless they represent theoretically or empirically significant subgroups (42, 43). Therefore, the 3-class model was determined as the best fitting model.

**Table 2:** Model fit information for latent class analysis models fit to data.

Class	AIC	BIC	SABIC	Entropy	VLMR^a^	BLRT^a^	Profile prevalence (%)
2	4413.657	4471.717	4430.443	0.528	0.1386	0.1457	53.7/46.3
3^b^	4398.199	4487.522	4424.023	0.921	0.4608	0.4668	17.1/64.1/18.8
4	4391.779	4512.365	4426.642	0.771	0.4859	0.4906	63.0/7.5/16.2/13.4

AIC, Akaike information criterion; BIC, Bayesian information criterion; BLRT, Bootstrapped likelihood ratio test; SABIC, Sample-size adjusted Bayesian information criterion; VLMR, Vuong-Lo–Mendell–Rubin.

a Chi-square statistic for the VLMR and the BLRT, when non-significant (*p* > 0.05), the VLMR and the BLRT test provide evidence that K-1 class model fits the data better than the K-class model.

b 3-class model was selected, based on its having the smallest SABIC and the largest entropy (>0.8).

### 3.3 Latent classes for lifestyle risk behaviors

**Figure 2:**
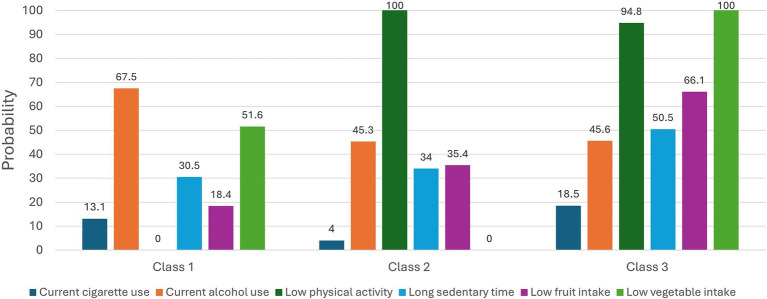
Latent classes of lifestyle risk behaviors for cardiovascular disease (CVD) among family caregivers. The figure shows the probabilities of each lifestyle risk behavior for CVD conditional on latent class membership. The proportions of each class: Class 1 (17.1%), Class 2 (18.8%), and Class 3 (64.1%). Class 1 was characterized by *physically active caregivers*, Class 2 by *physically inactive but healthy eaters*, and Class 3 by *physically inactive and unhealthy eaters*.

For the 3-class solution, conditional item-response probabilities are presented in Figure 2. A total of 17.1% of the sample was expected to belong to Class 1, which had a response pattern characterized by “*Physically active caregivers*.” Class 1 consisted of caregivers who had the highest probability for current alcohol drinkers (67.5%) and the lowest probabilities for low physical activity levels (0%), sedentary lifestyle (30.5%), and low fruit consumption (18.4%). Caregivers expected to belong to Class 2 (18.8%), characterized by “*Physically inactive, healthy eaters*.” Class 2 membership had the highest probability of low physical activity levels (100%) but had the lowest probabilities for current cigarette use (4%) and low vegetable consumption (0%). Class 3 constituted the largest latent class (64.1%) and was characterized by “*Physically inactive and unhealthy eaters*.” Class 3 members have a high probability for low physical activity levels (94.8%) and the highest probabilities for sedentary lifestyle (50.5%) and low fruit (66.1%) and vegetable consumption (100%).

### 3.4 Factors associated with identified class membership

In the unadjusted multinomial logistic regression models of the identified latent classes, age ≥65 years was associated with membership in Class 2, with Class 1 as the reference group (Table 3). Additionally, perceived financial difficulties, psychological distress, low self-efficacy in health management, poor sleep quality, dementia care, and caregiving for ≥20 h per week were associated with membership in Class 3. As there were no high correlations (coefficients range from 0.09 to 0.30) and multicollinearity (all VIFs < 1.2) among these significant factors, all the variables were included in the adjusted multinomial regression model. In the adjusted model, psychological distress remained significant (Table 4). Caregivers reporting psychological distress were more likely to belong to Class 3 rather than Class 1, compared to their counterpart without psychological distress. The fit of the model to the data improved when the predictor variables were added to the intercept-only model (X^2^ (18) = 58.25, Nagelkerke *R^2^* = 0.122, *p* < 0.001), and the Chi-Square Goodness-of-Fit test was not statistically significant, which indicates that the model is a good fit for the data (Pearson X^2^ (654) = 346.75, *p* = 0.206).

**Table 3:** Unadjusted multinominal logistic regression of class membership (reference group = Class 1).

	Class 2		Class 3	
	Β (SE)	OR (95% CI)	Β (SE)	OR (95% CI)
Age, years				
18–49	Ref.		Ref.	
50–64	0.786 (0.382)	2.194 (0.744, 5.226)	0.504 (0.333)	1.655 (0.859, 3.190)
≥ 65	0.679 (0.495)	**1.972 (1.034, 4.655)**	0.575 (0.414)	1.777 (0.786, 4.018)
Sex				
Male	Ref.		Ref.	
Female	0.524 (0.396)	1.690 (0.774, 3.688)	−0.140 (0.295)	0.870 (0.487, 1.554)
Race/ethnicity				
Non-Hispanic white	Ref.		Ref.	
Non-white^a^	−0.447 (0.462)	0.693 (0.257, 1.588)	0.293 (0.383)	1.340 (0.631, 2.848)
Education				
< College	−0.110 (0.419)	0.896 (0.393, 2.045)	0.171 (0.336)	1.187 (0.612, 2.301)
≥ College	Ref.		Ref.	
Marital status				
No married/partnered	Ref.		Ref.	
Married/partnered	0.330 (0.393)	1.391 (0.641, 3.018)	0.188 (0.313)	1.207 (0.652, 2.237)
Household income				
< $50,000	0.101 (0.487)	1.106 (0.424, 2.884)	0.529 (0.407)	1.697 (0.762, 3.779)
$50,000 - $99,999	−0.282 (0.448)	0.754 (0.312, 1.823)	−0.090 (0.365)	0.914 (0.445, 1.874)
≥ $100,000	Ref.		Ref.	
Perceived financial status				
Living comfortably	Ref.		Ref.	
Getting by	0.031 (0.441)	1.031 (0.433, 2.456)	−0.096 (0.367)	0.909 (0.441, 1.873)
Finding it difficult/very difficult	0.273 (0.489)	1.314 (0.502, 3.443)	0.901 (0.419)	**2.461 (1.079, 5.614)**
Rural–urban status				
Rural	Ref.		Ref.	
Urban	1.237 (0.652)	3.445 (0.954, 12.438)	0.695 (0.473)	2.003 (0.789, 5.087)
Body mass index				
<30 kg/m^2^	Ref.		Ref.	
≥30 kg/m^2^	0.237 (0.409)	1.267 (0.567, 2.835)	−0.138 (0.345)	0.871 (0.441, 1.719)
Medical conditions				
0–1 condition	Ref.		Ref.	
2–5 conditions	0.102 (0.425)	1.108 (0.479, 2.560)	−0.255 (0.345)	0.775 (0.393, 1.531)
Self-rated health				
Excellent /very good/ /good	Ref.		Ref.	
Fair/poor	0.724 (0.677)	2.063 (0.544, 7.824)	−0.546 (0.529)	0.579 (0.204, 1.641)
Psychological distress				
No distress	Ref.		Ref.	
Mild to severe distress	0.598 (0.445)	1.819 (0.756, 4.373)	1.285 (0.333)	**3.615 (1.878, 6.961)**
Self-efficacy				
High	Ref.		Ref.	
Low	0.442 (0.503)	1.556 (0.578, 4.191)	0.848 (0.397)	**2.336 (1.068, 5.105)**
Sleep duration				
< 7 h/night	−0.125 (0.393)	0.883 (0.407, 1.917)	−0.442 (0.316)	0.643 (0.345, 1.199)
≥ 7 h/night	Ref.		Ref.	
Sleep quality				
Good	Ref.		Ref.	.
Poor	0.534 (0.536)	1.706 (0.593, 4.907)	1.206 (0.365)	**3.341 (1.629, 6.851)**
Dementia care				
No	Ref.		Ref.	
Yes	0.540 (0.417)	1.271 (0.560, 2.888)	0.849 (0.308)	**2.336 (1.273, 4.289)**
Spousal caregiver				
No	Ref.		Ref.	
Yes	0.226 (0.506)	1.253 (0.463, 3.393)	0.210 (0.455)	1.233 (0.504, 3.020)
Caregiving hours				
< 20 h/week	Ref.		Ref.	
≥ 20 h/week	0.340 (0.377)	1.405 (0.669, 2.953)	0.767 (0.292)	**2.153 (1.211, 3.829)**

B, regression coefficient; CI, confidence interval; OR, odds ratio; Ref, reference; SE, standard error. Class 1 (*n* = 110) was characterized by physically active caregivers, Class 2 (*n* = 121) by physically inactive but healthy eaters, and Class 3 (*n* = 412) by physically inactive and unhealthy eaters. Bold font indicates statistical significance.

a The non-white category includes Black or African American, American Indian or Alaska Native, Asian, Native Hawaiian or other Pacific Islander, and Hispanic or Latino.

**Table 4:** Adjusted multinominal logistic regression of class membership (reference group = Class 1).

	Class 2		Class 3	
	Β (SE)	OR (95% CI)	Β (SE)	OR (95% CI)
Perceived financial status				
Living comfortably	Ref.		Ref.	
Getting by	−0.046 (0.447)	0.955 (0.396, 2.303)	−0.101 (0.350)	0.904 (0.453, 1.802)
Finding it difficult/very difficult	0.006 (0.564)	1.006 (0.331, 3.059)	0.310 (0.470)	1.363 (0.540, 3.444)
Psychological distress				
Minimal symptoms	Ref.		Ref.	
Mild to severe symptoms	0.409 (0.477)	1.467 (0.573, 3.757)	0.764 (0.354)	**2.173 (1.085, 4.350)**
Self-efficacy				
Completely/very confident	Ref.		Ref.	
Somewhat/a little/not confident	0.473 (0.473)	1.604 (0.632, 4.069)	0.550 (0.363)	1.733 (0.848, 3.542)
Sleep quality				
Good	Ref.		Ref.	
Poor	0.270 (0.623)	1.308 (0.384, 4.464)	0.732 (0.432)	2.079 (0.887, 4.873)
Dementia care				
No	Ref.		Ref.	
Yes	0.015 (0.500)	1.015 (0.379, 2.720)	0.706 (0.370)	2.026 (0.977, 4.202)
Caregiving hours				
< 20 h/week	Ref.		Ref.	
≥ 20 h/week	0.250 (0.387)	1.284 (0.599, 2.749)	0.598 (0.306)	1.819 (0.995, 3.324)

Age and sex controlled for. B = regression coefficient; CI = confidence interval; OR = odds ratio; Ref = reference; SE = standard error. Class 1 (*n* = 110) was characterized by physically active caregivers, Class 2 (*n* = 121) by physically inactive but healthy eaters, and Class 3 (*n* = 412) by physically inactive and unhealthy eaters. Bold font indicates statistical significance.

## 4 Discussion

This study identified three distinct classes of caregivers based on multiple lifestyle risk behaviors among a nationally representative sample of caregivers in the U.S. Notably, the majority (64.1%) were in the class characterized by the greatest number of lifestyle risk behaviors for CVD (i.e., low physical activity, prolonged sedentary time, low fruit and vegetable intake). Caregivers with psychological distress were more likely to fall into this class of “*physically inactive and unhealthy eaters*.” These findings underscore the importance of CVD prevention strategies tailored to subgroups and corresponding lifestyle risk behavior profiles in this at-risk population.

To our knowledge, this study is among the few that have investigated the co-occurrence patterns of lifestyle risk behaviors among caregivers, using established guidelines for each behavior through LCA. This approach enabled us to assess caregivers’ adherence to recommended levels of health behaviors, providing valuable information for providers working with this population. We found that insufficient physical activity (75.4%) and inadequate vegetable intake (71.2%) were the most prevalent risk behaviors, supporting previous findings that caregivers struggle with exercise and healthy eating due to time constraints (4, 7, 44). A previous study using the Behavioral Risk Factor Surveillance System data reported that 47–53% of caregivers met the recommended level of the aerobic activity, and 27–32% met the muscle-strengthening recommendation (45). Our criteria for physical activity, which are stricter by including both aerobic and muscle-strengthening activities as recommended, resulted in a higher proportion of non-adherent caregivers in the current study. These both studies indicate that over half of caregivers have low levels of physical activity and that only 14–18% of caregivers met dietary recommendations for fruit and vegetables intake (45), highlighting the need for improvement in caregivers’ physical activity and diets. Given the robust evidence linking recommended physical activity levels and vegetable intake with favorable outcomes in CVD mortality and morbidity (46–48), improving these areas is crucial to enhance cardiovascular health among caregivers.

Each identified class exhibited unique behavior configurations. Notably, over 60% of caregivers were characterized collectively by low levels of physical activity, prolonged sedentary time, and low levels of fruit and vegetable intake, while the other two classes were defined by one or two dominant lifestyle risk behaviors. This co-occurring pattern aligns with previous studies in general populations, although the proportion of the class with multiple unhealthy behaviors was higher in the current study of caregivers. For instance, a study of 10,638 Australians found that high-risk lifestyle behaviors, including poor diet quality (i.e., fruit/vegetable intake, soft drink and fast food consumption), physical inactivity, and excessive sitting, co-occurred in 33–40% of the sample, alongside excessive alcohol use and smoking (49). Similarly, a study in the Netherlands identified that 13.2% of the sample exhibited physical inactivity and unhealthy diet, along with current smoking and moderate alcohol consumption (50). In both studies, these behaviors also co-occurred at the favorable end of the spectrum, with a class characterized by low risk or healthy lifestyle behaviors (49, 50). Despite the limitation in making direct comparisons due to different measurements and criteria for the behaviors, it is noteworthy that no class in the current study was free of lifestyle risk behaviors, suggesting significant challenges for caregivers in maintaining healthy behaviors.

Consistent with previous studies (14, 26, 50–52), our findings indicate that caregivers experiencing psychological distress (i.e., depression, anxiety) are more likely to engage in multiple lifestyle risk behaviors, compared to those without such distress. A study of cancer caregivers found that higher levels of caregiver burden and perceived stress were associated with lower engagement in health-promoting behaviors (i.e., health responsibility, physical activity, nutrition, spiritual growth, interpersonal relationships, and stress management) (26). More broadly, risk behaviors, such as smoking, excessive drinking, physical inactivity, and unhealthy diet have been closely linked to poor mental health outcomes (50). In the caregiving context, particularly, psychological distress stemming from caregiving demands may contribute to the adoption of unhealthy behaviors as maladaptive coping mechanisms (53, 54). However, this relationship may be bidirectional. Unhealthy behaviors can also exacerbate psychological distress, potentially creating a self-reinforcing cycle (55). Longitudinal studies in the general population suggest that improvements in health behaviors, such as increased physical activity and greater fruit and vegetable intake, may reduce the risk of developing psychological distress (56, 57). On the other hand, in caregiving-specific research, one longitudinal study found that longer caregiving hours predicted poorer health behaviors, but psychological distress and burden did not have additional influences on health behaviors (7). This finding suggests that time constraints, rather than emotional strain, may be a more immediate barrier to engaging in healthy behaviors. Taken together, these findings highlight the complex and potentially reciprocal relationship between psychological distress and lifestyle behavioral patterns in caregivers. To better understand the directionality and underlying mechanisms of this relationship, more longitudinal research, particularly studies that track changes in mental health and lifestyle behaviors over the course of the caregiving trajectory, is needed.

This study underscores the importance of CVD prevention interventions that target co-occurring lifestyle risk behaviors among caregivers. The combined impact of multiple risk behaviors on chronic diseases, including CVD, and mortality, is significantly greater than that of individual behaviors alone (58, 59). A holistic approach to modifying multiple health behaviors, rather than addressing them in isolation, can maximize both the health benefits and cost-effectiveness of interventions (18). Importantly, our findings emphasize the need to address psychological factors when designing behavior change strategies for caregivers. Clinically, these findings suggest that healthcare providers should routinely screen for both psychological distress and lifestyle risk behaviors during clinical encounters. Tailored interventions based on the caregiver’s psychological and behavioral profile may be especially effective. For instance, caregivers who exhibit multiple unhealthy behaviors alongside high psychological distress may benefit most from integrated interventions that combine behavioral counseling with psychological support. In contrast, caregivers who maintain relatively healthy behaviors but experience elevated stress may benefit from preventive strategies focused on effective stress management, expanding coping skills, and peer support to sustain resilience and prevent behavioral decline. There is growing interest in mindfulness, mind–body, and positive psychological interventions that focus on emotional and cognitive processes (60). These interventions have shown promise in improving health behaviors, such as physical activity, diet, and medication adherence, and may also contribute to better cardiovascular outcomes (60). Future research should explore the implementation and effectiveness of such interventions in caregivers.

Beyond psychological distress, other health- and caregiving-related factors, including perceived financial difficulties, low self-efficacy in health management, poor sleep quality, dementia care responsibilities, and longer caregiving hours, were associated with membership of the “*Physically inactive, unhealthy eaters*” class, the unhealthiest behavior group in the unadjusted models. These factors should also be considered in identifying high-risk caregivers and designing targeted interventions. Particularly, self-efficacy plays a critical role in health behavior engagement. If one feels confident in their ability to engage in health behaviors, they are likely to commit to their goals and overcome emotional barriers, such as fear of failure (61). Additionally, successfully performing a behavior can further enhance self-efficacy (61). Therefore, improving self-efficacy through achievable goal setting, feedback, and skill-building may be especially effective, given caregivers’ challenges in prioritizing their own health needs. Caregiving experiences are dynamic and influenced by various interpersonal and environmental factors, such as the quality of the relationship with care recipients, the level of social support, access to caregiving resources, and cultural norms. These contextual factors may also significantly affect caregivers’ capacity to engage in health-promoting behaviors. Therefore, future research should incorporate these dimensions into intervention development to ensure that strategies are comprehensive, context-specific, and responsive to the diverse needs of caregivers.

### 4.1 Strengths and limitations

Our study has several strengths. To understand patterns of lifestyle risk behaviors among caregivers, we utilized LCA, a person-centered approach that identifies unobserved homogeneous subgroups within a given population based on particular combinations of observed indicators (62). This method is considered superior to variable-centered approaches (e.g., confirmatory factor analysis, regression), which extract generalized trends that apply to all respondents, for evaluating a population with underlying heterogeneous constructs, such as health behaviors (27, 62). Additionally, we used data from a nationally representative sample of caregivers across various conditions, enhancing the generalizability of our findings. By including a wide range of indicators of lifestyle risk behaviors and sociodemographic, health-related, and caregiving-related characteristics, we comprehensively examined the co-occurrence patterns and identified subgroups at higher risk for lifestyle risk behaviors.

Several limitations should be considered when interpreting our findings. First, the cross-sectional study design limits our ability to infer causality and determine directionality. For instance, we cannot ascertain whether psychological distress leads to worse lifestyle risk behavior patterns or if these risk behavior patterns increase the risk for psychological distress. Second, although we utilized nationally representative data, the sample of caregivers included in the analysis was relatively small. Future studies should include larger sample sizes to replicate our findings and enhance the robustness of the results. Third, the use of LCA introduces the possibility of misclassification, as class membership is determined based on probabilistic estimates. This means that individuals may be classified into a latent class even when their behavior patterns are not strongly distinct from those in other classes. Additionally, when the probability of a particular indicator pattern does not vary substantially across classes, that indicator may have limited utility in distinguishing between them (63). For example, in the present study, current alcohol consumption emerged as a relatively non-discriminative indicator. Fourth, the reliance of self-reported survey data for health behaviors introduces potential recall and social-desirability biases, which may impact the validity of our study findings. Future research should consider employing objective measures of health behaviors (e.g., accelerometers) to mitigate these biases. Lastly, we did not include sleep (i.e., sleep duration and quality), which is another lifestyle risk factor for CVD, as an indicator of lifestyle risk behaviors due to poor model fit in initial attempts to incorporate sleep into the LCA modeling. Previous studies have shown that sleep patterns often do not align with other lifestyle behaviors (diet, alcohol consumption, smoking, and physical activity). For example, high-risk sleep patterns can coexist with otherwise healthy behaviors, and low-risk sleep patterns can coexist with high-risk behaviors (49). This suggests that sleep may represent a different construct from other health behaviors regarding the co-occurrence of behaviors. Sleep behavior is often considered less volitional than other health behaviors and is influenced by various intrinsic and extrinsic factors (e.g., emotional distress, daytime behaviors, and sleep environment) (49, 64). Further research is needed to determine the co-occurrence of sleep problems with other lifestyle risk behaviors.

## 5 Conclusion

Lifestyle risk behaviors may play a critical role in the adverse health outcomes experienced by caregivers. Our study contributes to the growing literature by identifying distinct co-occurrence patterns of CVD-related lifestyle risk behaviors among caregivers. Alarmingly, most caregivers in our sample did not meet recommended guidelines for multiple health behaviors, highlighting a critical area for intervention. These findings underscore the need for holistic, multi-faceted lifestyle approaches that simultaneously address interconnected behaviors. Interventions that integrate mental health support with behavior change strategies may be particularly effective in promoting cardiovascular health in this at-risk population. Future research should explore the longitudinal impact of unhealthy behavioral patterns on cardiovascular outcomes among caregivers, as well as the mechanisms linking psychological distress and lifestyle behaviors.

## Data Availability

Publicly available datasets were analyzed in this study. This data can be found here: https://hints.cancer.gov/data/download-data.aspx.
