# Supplementary material for: Patterns of lifestyle risk behaviors for cardiovascular disease in family caregivers: a latent class analysis
Source: Front Public Health. 2025 Jun 17;13:1593898. doi: 10.3389/fpubh.2025.1593898 (PMC12209217; doi:10.3389/fpubh.2025.1593898)
Supplement: Supplementary file 1 [file Data_Sheet_1.docx]

Supplementary Material

# Supplementary Tables

Supplementary Table 1.1. Sample Characteristics by the Identified Latent Classes (Data with Missing Values)

|  | Total | Class 1 | Class 2 | Class 3 | *p* |
| --- | --- | --- | --- | --- | --- |
|  |  | n = 110 | n = 121 | n = 412 |  |
|  |  | n (weighted %) | n (weighted %) | n (weighted %) |  |
| Age, years |  |  |  |  | .247 |
| 18-49 | 199 (42.4) | 39 (53.5) | 42 (34.5) | 118 (40.7) |  |
| 50-64 | 253 (42.0) | 45 (34.3) | 46 (48.7) | 162 (42.9) |  |
| ≥ 65 | 182 (15.6) | 25 (12.2) | 30 (16.9) | 127 (16.4) |  |
| Sex |  |  |  |  | .135 |
| Male | 223 (40.4) | 43 (40.3) | 27 (27.5) | 153 (44.1) |  |
| Female | 381 (59.6) | 58 (59.7) | 85 (72.5) | 238 (55.9) |  |
| Race/ethnicity |  |  |  |  | .122 |
| Non-Hispanic white | 367 (62.0) | 71 (60.0) | 64 (47.8) | 232 (67.0) |  |
| Non-white^a^ | 217 (38.0) | 30 (40.0) | 44 (52.2) | 143(33.0) |  |
| Education |  |  |  |  | .630 |
| < College | 289 (65.8) | 41 (63.7) | 45 (61.2) | 203 (67.9) |  |
| ≥ College graduate | 344 (34.2) | 66 (36.3) | 74 (38.8) | 204 (32.1) |  |
| Marital status |  |  |  |  | .704 |
| Married/partnered | 428 (72.9) | 70 (69.3) | 85 (76.0) | 273 (73.3) |  |
| No married/partnered | 199 (27.1) | 35 (30.7) | 32 (24.0) | 132 (26.7) |  |
| Household income |  |  |  |  | .443 |
| < $50,000 | 233 (34.6) | 30 (26.1) | 35 (31.1) | 168 (38.4) |  |
| $50,000 - $99,999 | 203 (32.7) | 31 (39.1) | 37 (30.6) | 135 (31.1) |  |
| ≥ $100,000 | 198 (32.7) | 47 (34.8) | 45 (38.3) | 106 (30.5) |  |
| Perceived financial status |  |  |  |  | .107 |
| Living comfortably | 247 (38.1) | 56 (41.8) | 47 (39.6) | 144 (36.4) |  |
| Getting by | 218 (36.8) | 31 (44.0) | 43 (41.7) | 144 (33.0) |  |
| Finding it difficult | 143 (25.1) | 16 (14.2) | 22 (18.6) | 105 (30.6) |  |
| Rural-urban status |  |  |  |  | .117 |
| Urban | 578 (88.7) | 100 (81.3) | 112 (93.7) | 366 (89.7) |  |
| Rural | 65 (11.3) | 10 (18.7) | 9 (6.3) | 46 (10.3) |  |
| Body mass index |  |  |  |  | .502 |
| <30kg/m^2^ | 382 (62.3) | 80 (63.6) | 74 (69.1) | 228 (59.8) |  |
| ≥30kg/m^2^ | 251 (37.7) | 29 (36.4) | 42 (30.9) | 180 (40.2) |  |
| Medical conditions |  |  |  |  | .499 |
| 0-1 condition | 430 (71.2) | 83 (74.2) | 83 (76.0) | 264 (68.7) |  |
| 2-5 conditions | 200 (28.8) | 24 (25.8) | 32 (24.0) | 144 (31.3) |  |
| Self-rated health |  |  |  |  | .061 |
| Excellent/very good/good | 544 (84.0) | 98 (87.2) | 112 (94.2) | 334 (79.9) |  |
| Fair/poor | 95 (16.0) | 11 (12.8) | 8 (5.8) | 76 (20.1) |  |
| Psychological distress |  |  |  |  | <.001 |
| No distress | 433 (68.2) | 89 (84.6) | 97 (77.1) | 247 (59.8) |  |
| Mild to severe distress | 196 (31.8) | 19 (15.4) | 21 (22.9) | 156 (40.2) |  |
| Self-efficacy |  |  |  |  | .062 |
| High | 453 (67.8) | 92 (79.6) | 86 (71.5) | 275 (62.6) |  |
| Low | 190 (32.2) | 18 (20.4) | 35 (28.5) | 137 (37.4) |  |
| Sleep duration |  |  |  |  | .214 |
| < 7 hours/night | 301 (45.8) | 42 (38.8) | 53 (39.2) | 206 (50.1) |  |
| ≥ 7 hours/night | 327 (54.2) | 66 (61.2) | 64 (60.8) | 197 (49.9) |  |
| Sleep quality |  |  |  |  | .016 |
| Good | 469 (71.5) | 93 (85.8) | 90 (77.6) | 286 (64.6) |  |
| Poor | 166 (28.5) | 17 (14.2) | 29 (22.4) | 120 (35.4) |  |
| Dementia care |  |  |  |  | .020 |
| Yes | 183 (26.8) | 28 (16.8) | 32 (20.5) | 123 (32.1) |  |
| No | 460 (73.2) | 82 (83.2) | 89 (79.5) | 289 (67.9) |  |
| Spousal caregiver |  |  |  |  | .822 |
| Yes | 160 (20.5) | 24 (23.3) | 32 (19.5) | 104 (19.8) |  |
| No | 483 (79.5) | 86 (76.7) | 89 (80.5) | 308 (80.2) |  |
| Caregiving hours |  |  |  |  | .041 |
| < 20 hours/week | 349 (59.0) | 71 (70.4) | 75 (64.8) | 203 (53.0) |  |
| ≥ 20 hours/week | 233 (41.0) | 32 (29.6) | 32 (35.2) | 169 (47.0) |  |

^a^The non-white category includes Black or African-American, American Indian or Alaska Native, Asian, Native Hawaiian or other Pacific Islander, Hispanic or Latino.

Supplementary Table 1.2. Sample Characteristics by the Identified Latent Classes (Imputed Data)

|  | Total | Class 1 | Class 2 | Class 3 | *p* |
| --- | --- | --- | --- | --- | --- |
|  |  | n (weighted %) | n (weighted %) | n (weighted %) |  |
| Age, years |  |  |  |  | .229 |
| 18-49 | 4231 (42.2) | 826 (53.4) | 906 (34.9) | 2499 (40.4) |  |
| 50-64 | 5399 (42.3) | 951 (34.3) | 995 (49.2) | 5399 (42.3) |  |
| ≥ 65 | 3864 (15.6) | 532 (12.3) | 637 (15.9) | 3864 (15.6) |  |
| Sex |  |  |  |  | .132 |
| Male | 4919 (39.9) | 955 (40.0) | 632 (28.3) | 3332 (43.4) |  |
| Female | 8545 (60.1) | 1346 (60.0) | 1900 (71.7) | 5299 (56.6) |  |
| Race/ethnicity |  |  |  |  | .137 |
| Non-Hispanic white | 8437 (61.9) | 1599 (59.8) | 1513 (48.8) | 5325 (66.6) |  |
| Non-white^a^ | 5007 (38.1) | 702 (40.2) | 1015 (51.2) | 3290 (33.4) |  |
| Education |  |  |  |  | .656 |
| < College | 6165 (65.6) | 897 (63.7) | 966 (61.1) | 4302 (67.6) |  |
| ≥ College graduate | 7328 (34.4) | 1410 (36.3) | 1573 (38.9) | 4345 (32.4) |  |
| Marital status |  |  |  |  | .694 |
| Married/partnered | 9180 (72.8) | 1532 (69.2) | 1834 (75.8) | 5814 (73.1) |  |
| No married/partnered | 4307 (27.2) | 773 (30.8) | 703 (24.2) | 2831 (26.9) |  |
| Household income |  |  |  |  | .421 |
| < $50,000 | 4945 (34.4) | 635 (25.9) | 759 (30.8) | 3551 (38.4) |  |
| $50,000 - $99,999 | 4328 (32.9) | 668 (39.1) | 801 (31.7) | 2859 (31.2) |  |
| ≥ $100,000 | 4221 (32.7) | 1005 (35.0) | 977 (37.5) | 2239 (30.5) |  |
| Perceived financial status |  |  |  |  | .068 |
| Living comfortably | 5427(38.0) | 1237 (42.3) | 1071 (39.9) | 3119 (36.0) |  |
| Getting by | 4859 (36.8) | 692 (43.0) | 973 (41.8) | 3194 (33.2) |  |
| Finding it difficult | 3182 (25.1) | 374 (14.7) | 488 (18.3) | 2320 (30.8) |  |
| Rural-urban status |  |  |  |  | .117 |
| Urban | 12138 (88.7) | 2100 (81.3) | 2352 (93.7) | 7686 (89.7) |  |
| Rural | 1365 (11.3) | 210 (18.7) | 189 (6.3) | 966 (10.3) |  |
| Body mass index |  |  |  |  | .531 |
| <30kg/m^2^ | 8144 (62.2) | 1686 (63.2) | 1616 (68.5) | 4842 (59.9) |  |
| ≥30kg/m^2^ | 5349 (37.8) | 623 (36.8) | 920 (31.5) | 3806 (40.1) |  |
| Medical conditions |  |  |  |  | .500 |
| 0-1 condition | 9184 (71.0) | 1776 (73.8) | 1826 (75.7) | 5582 (68.6) |  |
| 2-5 conditions | 4306 (29.0) | 531 (26.2) | 709 (24.3) | 3066 (31.4) |  |
| Self-rated health |  |  |  |  | .056 |
| Excellent/very good/good | 11464 (83.9) | 2078 (87.2) | 2353 (93.4) | 7033 (79.8) |  |
| Fair/poor | 2035 (16.1) | 231 (12.8) | 187 (6.6) | 1617 (20.2) |  |
| Psychological distress |  |  |  |  | <.001 |
| No distress | 9256 (67.5) | 1892 (84.2) | 2071 (74.5) | 5293 (59.6) |  |
| Mild to severe distress | 4233 (32.5) | 416 (15.8) | 467 (25.5) | 3350 (40.4) |  |
| Self-efficacy |  |  |  |  | .062 |
| High | 9513 (67.8) | 1932 (79.6) | 1806 (71.5) | 5775 (62.6) |  |
| Low | 3990 (32.2) | 378 (20.4) | 735 (28.5) | 2877 (37.4) |  |
| Sleep duration |  |  |  |  | .298 |
| < 7 hours/night | 6500 (46.5) | 908 (39.3) | 1160 (42.3) | 4432 (50.2) |  |
| ≥ 7 hours/night | 4988 (53.5) | 1400 (60.7) | 1377 (57.7) | 4211 (49.8) |  |
| Sleep quality |  |  |  |  | .010 |
| Good | 9955 (71.5) | 1953 (85.8) | 1927 (78.1) | 6075 (64.5) |  |
| Poor | 3540 (28.5) | 357 (14.2) | 612 (21.9) | 2571 (35.5) |  |
| Dementia care |  |  |  |  | .020 |
| Yes | 3843 (26.8) | 588 (16.8) | 672 (20.5) | 2583 (32.1) |  |
| No | 9660 (73.2) | 1722 (83.2) | 1869 (79.5) | 6069 (67.9) |  |
| Spousal caregiver |  |  |  |  | .822 |
| Yes | 3360 (20.5) | 504 (23.3) | 672 (19.5) | 2184 (19.8) |  |
| No | 10143 (79.5) | 1806 (76.7) | 1869 (80.5) | 6468 (80.2) |  |
| Caregiving hours |  |  |  |  | .019 |
| < 20 hours/week | 7996 (57.9) | 1589 (70.2) | 1732 (62.6) | 4675 (52.2) |  |
| ≥ 20 hours/week | 5446 (42.0) | 714 (29.8) | 795 (37.4) | 3937 (47.8) |  |

^a^The non-white category includes Black or African-American, American Indian or Alaska Native, Asian, Native Hawaiian or other Pacific Islander, Hispanic or Latino.
